# Bilateral Adrenal Hemorrhage in a Patient with Myelodysplastic Syndrome: Value of MRI in the Differential Diagnosis

**DOI:** 10.1155/2013/479836

**Published:** 2013-12-05

**Authors:** Lucia Manganaro, Najwa Al Ansari, Flavio Barchetti, Matteo Saldari, Claudia Vitturini, Marianna Glorioso, Valeria Buonocore, Giovanni Barchetti, Francesca Maccioni

**Affiliations:** Department of Radiological Sciences, Oncology and Pathology, University of Rome, Sapienza, Italy

## Abstract

Bilateral adrenal hemorrhage is a rare potentially life-threatening event that occurs either in traumatic or nontraumatic conditions. The diagnosis is often complicated by its nonspecific presentation and its tendency to intervene in stressful critical illnesses. Due to many disorders in platelet function, hemorrhage is a major cause of morbidity and mortality in patients affected by myeloproliferative diseases. We report here the computed tomography and magnetic resonance imaging findings of a rare case of bilateral adrenal hemorrhage in a patient with myelodysplastic syndrome, emphasizing the importance of MRI in the differential diagnosis.

## 1. Introduction 

Acute bilateral adrenal hemorrhage is an extremely rare disorder and it is difficult to diagnose because of its nonspecific presentation [1]. This condition frequently occurs in association with an extreme physical stress and may lead to acute adrenal insufficiency or death if not promptly and properly treated [2]. In adults, major causes of bilateral adrenal hemorrhage are the presence of meningococcemia or sepsis from any organism [3], underlying adrenal tumors, burns, or hypotension related to hemorrhagic diathesis, especially the one caused by uncontrolled anticoagulation [4] in patients treated with heparin, warfarin, or direct thrombin inhibitors. We report a rare case of acute bilateral adrenal hemorrhage in a patient affected by myelodysplastic syndrome.

## 2. Case Report

A 65-year-old man affected by myelodysplastic syndrome, but not already treated, was admitted to the emergency room with suspected pancreatitis. He complained of dyspnea, nausea, vomit, and acute epigastric pain with radiation to the back. There was no history of trauma or anticoagulant therapy and he denied fever, hematuria, and urinary symptoms and blood pressure was 110/80 mmHg. Laboratory tests revealed a platelet count of 82,000/mm^3^, prolonged INR (International Normalized Ratio) and aPTT (activated partial thromboplastin time), hyponatremia, hyperkalemia, and anemia, normal serum electrolytes, lipase, and amylase levels.

**Figure 1:**
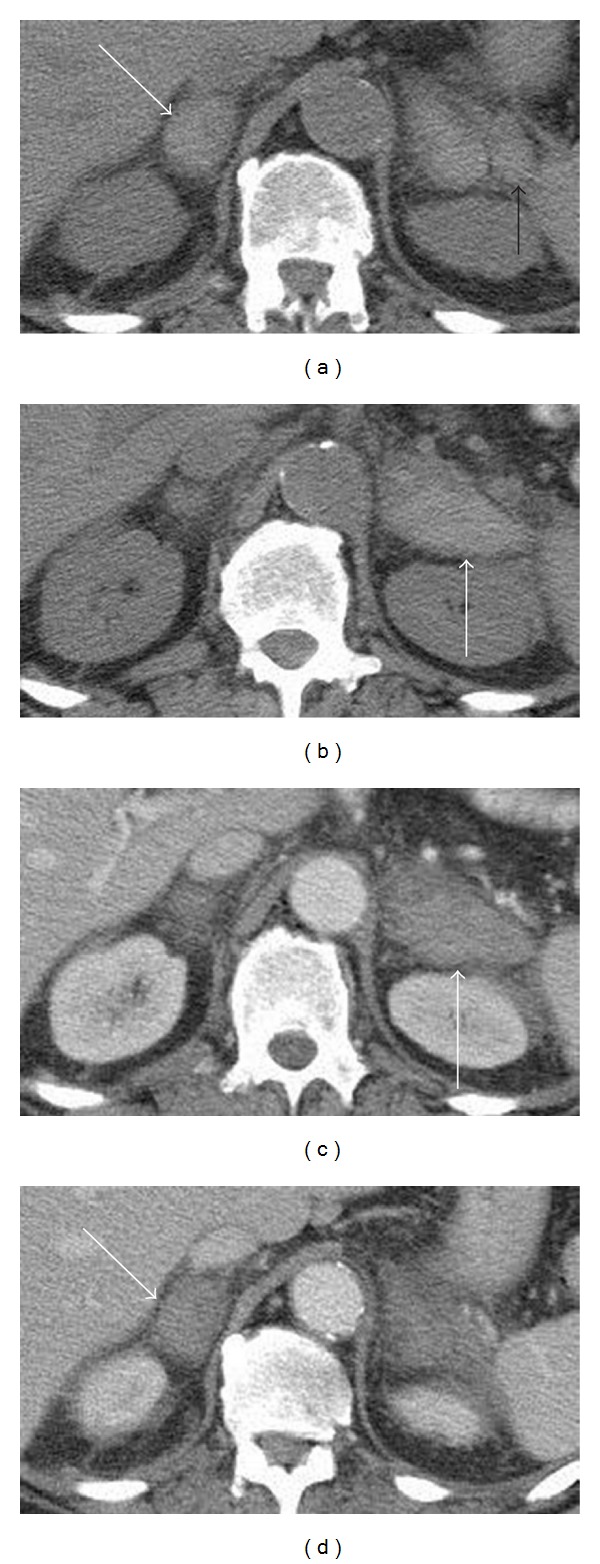
Axial non-contrast-enhanced CT image (a) shows bilateral adrenal enlargement (white arrows) with high attenuation values. Axial contrast-enhanced CT image (b) shows a bilateral homogeneously enhancing adrenal mass with solid appearance. Note the stranding of the periadrenal fat (black arrow) and the lack of regional lymphadenopathy.

A multislice computed tomography (MSCT) scan (Light-speed GE Medical Systems, Milwaukee, WI, USA) of the chest, abdomen, and pelvis was performed with administration of 110 mL of intravenous contrast agent (Iomeron 350, Bracco, Milan, Italy) at a speed of 4.0 mL/s. The exam revealed bilateral and symmetric enlargement of the adrenal glands which were round and with hyperdense areas on precontrast scans (each gland attenuation values were of 55 Hounsfield Units on an average), consistent with recent bleeding. Mild peripheral heterogeneous enhancement on the arterial phase, stranding of the periadrenal fat, and pleural effusion were also detected, whereas there was no evidence of lymphadenopathy (Figure 1). The patient was treated with blood transfusions and a prompt adrenal hormone replacement therapy, with glucocorticoids combined with mineralocorticoids.

After one week, an abdominal magnetic resonance imaging (MRI) exam was performed to better evaluate the adrenal glands (Avanto 1.5T, Siemens HealthCare, Erlangen, Germany). Multiplanar (axial, coronal, and sagittal) T1- and T2-weighted with and without fat saturation, T1-weighted in-phase and out-of-phase spoiled gradient-recalled-echo (GRE), DWI, and ADC images were acquired.

**Figure 2:**
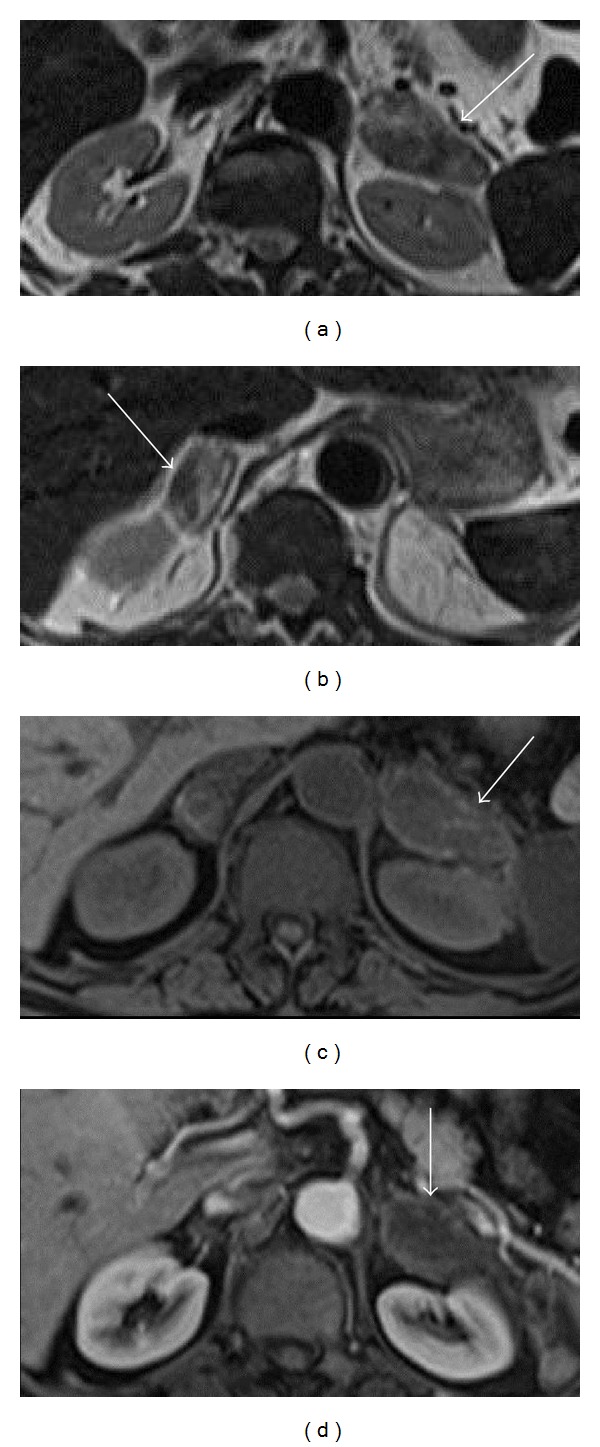
Bilateral enlarged adrenal glands with high signal intensity on axial T2- ((a), (b)) and T1-weighted (c) MRI images obtained before administration of gadolinium. Axial T1-weighted images (d) obtained after contrast injection reveal hyperintense masses with mild heterogeneous enhancement in hepatic arterial dominant phase.

**Figure 3:**
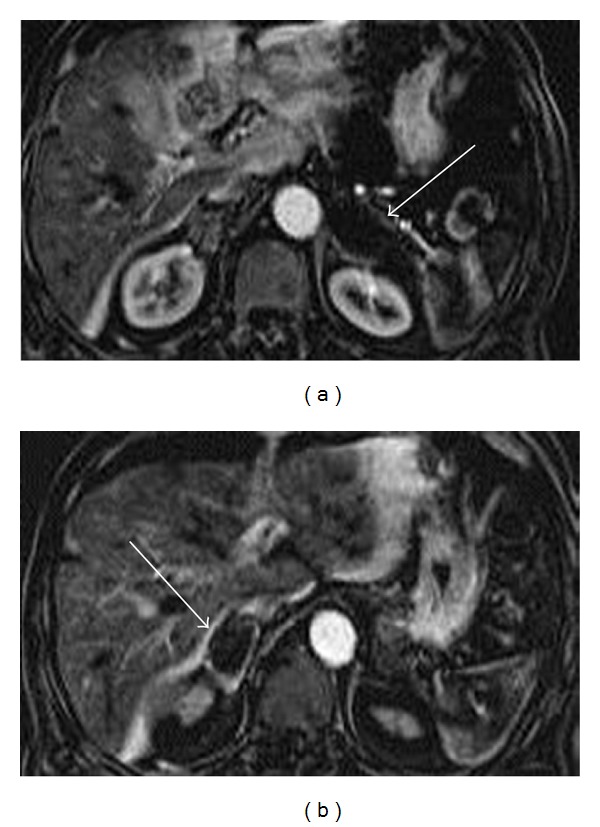
On axial contrast-enhanced subtracted MR images (a), (b) any signal from blood products is removed and the hematoma appears as a signal void (white arrows) because it does not contain enhancing elements, excluding the presence of a hemorrhagic neoplasm.

MRI revealed bilaterally enlarged adrenal glands: the diameter of the left adrenal gland was 65 mm and the diameter of the right adrenal gland was 40 mm. Heterogeneous hyperintensity, consistent with subacute hemorrhage, was detected on both T1- and T2-weighted images. The exam also showed a subtle signal loss on T1-weighted in-phase GRE images compared to the T1-weighted GRE out-of-phase images and a mild heterogeneous peripheral enhancement after contrast (MultiHance, Bracco Diagnostic, Milan, Italy) injection (Figure 2). Finally, on contrast-enhanced MR subtracted images the hematomas appeared as areas of signal void, excluding the presence of a hemorrhagic neoplasm (Figure 3).

## 3. Discussion

The exact incidence of adrenal hemorrhage in the general population is unknown, although some postmortem studies have found a prevalence of 0.14–1.1%. Many cases do not have specific signs of adrenal insufficiency, which usually occurs in patients with damage of more than 90% of the adrenal cortex, and the diagnosis is incidentally made on imaging performed for other reasons [5]. Unilateral adrenal hemorrhage can be usually managed conservatively, whereas bilateral presentation is often life-threatening and, due to its no specific clinical symptoms, it can only be diagnosed at the time of surgery or at postmortem examination [6]. The most common physical and laboratory findings are abdominal pain, fever, hypotension, confusion, decreased hemoglobin level (greater than 1.5 g/dL), and prolonged international normalized ratio (greater than 1.4). Survival from bilateral adrenal hemorrhage usually requires prompt adrenal hormone replacement therapy, either with glucocorticoids alone or glucocorticoids combined with mineralocorticoids.

The pathophysiology of bilateral adrenal haemorrhage is still unknown. However, some particular anatomic features of the glands, such as a rich arterial blood supply that feeds into a dense and delicate subcapsular capillary network through limited venules, predispose adrenal medulla to venous congestion and thrombosis. Stress, increasing corticotrophin secretion, produces a dramatic rise in blood flow and the risk of hemorrhagic infarction [7].

Although bilateral adrenal hemorrhage is classically associated with meningococcemia (Waterhouse-Friderichsen Syndrome), it may occur in sepsis from any organism [8]. Traumatic events, burns, pregnancy, antiphospholipid syndrome, heparin-associated thrombocytopenia, thrombophilic syndromes, anticoagulant therapy, and abdominal surgery are some of the other causes of adrenal bleeding [9–11].

Hematomas can also arise in the setting of preexisting adrenal neoplasms, such as pheochromocytoma, myelolipoma, melanoma, neuroblastoma, adrenocortical carcinoma, hepatocellular carcinoma, and lung cancer metastases and should be suspected when CT or MR imaging shows a hemorrhagic adrenal mass of heterogeneous attenuation or signal intensity that demonstrates enhancement [12–15].

Due to number and function platelet alterations, thrombotic and hemorrhagic diathesis represents an event which may occur in myelodysplastic syndromes (MDS) [16]. Dayyani et al. have studied the most common disease-related causes of death in patients with lower-risk myelodysplastic syndrome. Infection (38% of all deaths), transformation to AML (15%), and fatal hemorrhage (13%) constituted 96% of all known causes of MDS-related deaths. Hemorrhage occurred in the central nervous system in 26%, and gastrointestinal bleeding and pulmonary hemorrhage each was responsible for 24% of bleeding-related deaths [17].

To our knowledge, CT and MRI findings of bilateral adrenal bleeding in a patient affected by myelodysplastic syndrome have never been described before in literature.

Sacerdote et al. [18] proposed 5 patterns of adrenal hemorrhage CT appearance with the aim to differentiate hematomas from other adrenal pathologies, particularly neoplasms. In the first pattern the hemorrhage appears as a round or ovoid solid adrenal mass with an attenuation comparable to the soft tissue, as in our patient. With this CT findings, high density on precontrast scans and diminishing size or resolution of the adrenal masses are usually diagnostic of hematomas, whereas the presence of a rim of enhancing does not permit distinguishing adrenal bleeding from neoplasm [18–20]. In addition to this, all types of adrenal hemorrhages may liquefy, calcify, or become pseudocysts over time and should be evaluated further in suspected cases.

Although CT scan is the the most widespread used imaging modality in the emergency setting, MR is more sensitive and specific for diagnosing adrenal hemorrhage and determining if blood is the sole component of the hematoma. This finding may exclude the presence of an underlying neoplasm along with the presence or absence of enhancement after administration of gadolinium contrast material. Blood products appearance on MRI depends on their stage of evolution. They evolve over time into the different components of acute, subacute, and chronic hemorrhage (deoxyhemoglobin, methemoglobin, and hemosiderin) [12]. In acute bleeding (less than 7 days) deoxyhemoglobin appears isointense or hypointense on T1-weighted images and has low signal intensity on T2-weighted images. Subacute blood (7 days to 7 weeks) in the form of methemoglobin is hyperintense on T1-weighted images. Initially, methemoglobin is intracellular and has low signal intensity on T2-weighted images. Subsequently, as the red cells lyse and the methemoglobin becomes extracellular, it has high signal intensity on T2-weighted images. Chronic hematoma (more than 7 weeks) has low signal intensity on both T1- and T2-weighted images because of the presence of hemosiderin.

The differential diagnosis between simple adrenal hemorrhage and hemorrhage occurring in an underlying mass may be performed using contrast-enhanced MRI with subtraction imaging. On MRI, areas of high signal will be present on unenhanced T1-weighted sequences because of the presence of intracellular or extracellular methemoglobin. The presence of bright signal on unenhanced T1-weighted MRI sequences makes the qualitative evaluation of enhancement difficult. However, any signal from blood products will be removed on subtraction images and a simple hematoma should appear as a signal void because it will not contain enhancing elements, which may, on the other hand, be present in case of a hemorrhagic neoplasm [21].

In our patient, the association of MRI and CT was able to distinguish a hematoma from a hemorrhagic adrenal tumor avoiding the risks and discomfort of an open surgical procedure or biopsy, which may, on the contrary, be necessary in suspected cases.
